# The effect of local land use and loss of forests on bats and nocturnal insects

**DOI:** 10.1002/ece3.2160

**Published:** 2016-05-27

**Authors:** Julia T. Treitler, Olga Heim, Marco Tschapka, Kirsten Jung

**Affiliations:** ^1^ Evolutionary Ecology and Conservation GenomicsUniversity UlmUlmGermany; ^2^ Smithsonian Tropical Research InstituteBalboaPanama; ^3^ Present address: RG Ecology and Environmental EducationInstitute of Biology and ChemistryUniversity of HildesheimHildesheimGermany; ^4^ Present address: Leibniz Institute for Zoo‐ and Wildlife Research (IZW)BerlinGermany

**Keywords:** Agricultural areas, biodiversity, conservation, grasslands, landscape ecology, trophic interactions

## Abstract

Land‐use intensification at local and landscape level poses a serious threat to biodiversity and affects species interactions and ecosystem function. It is thus important to understand how interrelated taxa respond to land‐use intensification and to consider the importance of different spatial scales. We investigated whether and how local land‐use intensity and landscape features affect the predator–prey interaction of bats and insects. Bats and nocturnal insects were assessed on 50 grassland sites in the Schorfheide‐Chorin. We analyzed the effect of local land use and distance to forested areas as a proxy for site accessibility on bats and insects and their biological interaction measured in bat's feeding activity. Insect abundance increased with higher land‐use intensity, while size and diversity of insects decreased. In contrast, bat activity, diversity, and species composition were determined by the distance to forested areas and only slightly by land‐use intensity. Feeding attempts of bats increased with higher insect abundance and diversity but decreased with insect size and distance to forested areas. Finally, our results revealed that near forested areas, the number of feeding attempts was much lower on grassland sites with high, compared to those with low land‐use intensity. In contrast, far from forests, the feeding attempts did not differ significantly between intensively and extensively managed grassland sites. We conclude that the two interrelated taxa, bats and insects, respond to land‐use intensification on very different scales. While insects respond to local land use, bats are rather influenced by surrounding landscape matrix. Hereby, proximity to forests reveals to be a prerequisite for higher bat species diversity and a higher rate of feeding attempts within the area. However, proximity to forest is not sufficient to compensate local high land‐use intensity. Thus, local land‐use intensification in combination with a loss of forest remnants weakens the interaction of bats and insects.

## Introduction

A composition of managed agricultural areas (Matson et al. 1997) and remaining fragments of production forest systems are shaping the landscapes scenery of Central Europe today. About one‐third of these agricultural landscapes are dominated by grasslands (EUROSTAT 2011), which are considered to be highly diverse and complex ecosystems (McNaughton 1994). A large percentage of these grasslands, however, are currently intensively managed for hay production or cattle grazing. The intensification of grassland management during the last century, mainly manifested in an increase of fertilizer application, higher mowing frequency, and stocking rates, has led to a species decline in many taxa (Allan et al. 2014), including plants (Andreasen et al. 1996), invertebrates (Hendrickx et al. 2007), and vertebrates (Donald et al. 2001; Benton et al. 2002; Wickramasinghe et al. 2003). Higher land‐use intensity has also shown to weaken the associations between taxa via a loss in taxon diversities, leading to a breakdown of species interactions (Manning et al. 2015) necessary for the ecosystem services such as pollination or biological control (Tscharntke et al. 2005).

The structural composition of the landscape, however, can influence a variety of ecological responses at the local scale (Fahrig et al. 2011) and may compensate for local high‐intensity management (Tscharntke et al. 2005). Remaining key structures, maintaining or enhancing compositional habitat heterogeneity in agriculturally used landscapes, are forest fragments that provide food, shelter, breeding, and roosting opportunities for many animal species. Forest fragments and other landscape elements such as hedges and old growth trees (Heim et al. 2015) may even provide a source for species diversity within the landscape and could potentially facilitate recolonization of agricultural areas after disturbance events (Hendrickx et al. 2007). It is thus important to integrate information about the surrounding landscapes into local‐scale investigations of biodiversity in agricultural areas as it determines accessibility and potential habitat and resource diversity at larger scales (Dunning et al. 1992). Landscape composition and configuration can also have an important indirect effect on local species populations by directly affecting the presences of their predators or prey (Dunning et al. 1992).

Different species respond to environmental conditions at very different spatial scales (Chase 2014). This is to a large extent influenced by a species‐specific perception of the habitat (Tews et al. 2004) and can be reasoned by a species' degree of habitat and resource specialization (Öckinger et al. 2010) and mobility (Concepción et al. 2015). Species depending on very distinct environmental conditions and limited mobility are most likely the first to be affected by unfavorable local changes in land use. In contrast, highly mobile species integrate habitat conditions over a wider area and may easily escape and move toward remaining suitable areas.

Insectivorous bats are very mobile and opportunistic feeders (McCracken et al. 2012). Among them, the ground foraging *Myotis myotis* is known to respond to locally varying land management practices and profits from mowing events in grassland systems (Rainho et al. 2010). In general, however, species richness and activity of bats in agricultural areas increase with landscape heterogeneity and site accessibility (Fuentes‐Montemayor et al. 2011; Frey‐Ehrenbold et al. 2013), in proximity to trees (Lumsden and Bennett 2005), hedges (Boughey et al. 2011) and forest remnants (Kalda et al. 2014; Heim et al. 2015). On the contrary, abundance and species richness of insects, the nutritional resource for European bats, are strongly determined by local plant diversity and vegetation complexity (Haddad et al. 2000; Koricheva et al. 2000; Reid and Hochuli 2007). Previous studies on day‐active insects in grasslands revealed that increasing local land‐use intensity negatively affects species richness and the assemblage structure of insects by filtering out rare and specialized species with low mobility, thus leading to an increased dominance of already abundant and generalist species (Weiner et al. 2014; Simons et al. 2015). Thus, the combination of landscape composition and local land use (e.g., Tscharntke et al. 2005; Hendrickx et al. 2007) may affect the trophic interaction between bats and their prey.

Here, we investigated whether the relative abundance and species composition of bats and insects respond to land‐use changes at local and landscape scales. In addition, we examine whether feeding activity, used as a direct measure for bat–insect interaction, may be affected by a combined effect of both environmental scales. We then discuss whether this may have implications on the ecosystem service contribution, for example, herbivore insect control provided by bats in an agricultural landscape.

We expected that abundance, diversity, and size of insects are strongly determined by local land use, whereby insect abundance increases, while diversity and size of insects decrease with higher land‐use intensity. We predicted that bat activity, diversity, and species composition are predominantly determined by the proximity to forest remnants and less affected by local land‐use intensity. As bats are generally very opportunistic feeders, we expected increased feeding activity with higher resource abundance. However, we anticipated that a large distance to forested areas might hinder bats to reach potentially suitable foraging sites. We thus hypothesized that bat–insect interaction on managed grassland sites in agricultural landscapes to be affected by a combination of environmental changes at the local and the landscape scale.

## Materials and Methods

### Study area

Our study was conducted in the Biosphere Reserve Schorfheide‐Chorin (1300 km^2^) in the state of Brandenburg, in northeastern Germany. The Schorfheide‐Chorin is a young glacial landscape characterized by ground and terminal moraines, lakes, fens, and mires (Fischer et al. 2010). Mean annual temperature ranges between 8.0 and 8.5°C and mean precipitation between 500 and 600 mm. The Schorfheide‐Chorin is one of three “Biodiversity Exploratories” for long‐term and functional biodiversity research (www.biodiversity-exploratories.de) and harbors 50 permanently marked grassland sites (Fischer et al. 2010). Grassland sites are managed as pastures, mown pastures, or meadows on mineral (*N* = 23) and fen soils (*N* = 27) and differ in land‐use intensity and distance toward forested areas (maximum distance 500 m).

### Acoustic monitoring of bats

Acoustic monitoring of bats was conducted along a point‐stop transect (Jung et al. 2012) of 200 m around the outer borders of each grassland site (50 × 50 m). This assured the detection of bats with very patchy activity and low‐intensity echolocation calls (e.g., *Myotis*,* Plecotus*). Monitoring time at point stops and during transect walks (direct line between point stops) was 3 min resulting in a total survey time of 24 min per plot and visit. We are aware that these data only represent a snapshot of bat activity at each site. However, comparing species richness and relative activity obtained through these transects to those from automatic and stationary recording units operated synchronously at the center of each grassland site from sunset to 1:00 AM (Heim et al. 2015) did not reveal significant differences. Each grassland plot was sampled five times (once per month) between May and September 2010. Monitoring started 30 min after local sunset (Eberswalde‐Finow, Germany, 52°49′′N and 13°31′′E) and continued until 1:00 AM to account for the first peak of bat activity in the night (Rydell et al. 1996). Multiple plots (4–6) were sampled each night and sampling order of the plots was randomized for each sampling month. To control for the potential confounding effect of moonlight, we visited grassland sites only in a two‐week period just before and after new moon. Recording was aborted in case of rainfall and the survey was repeated in the following night.

Echolocation calls of bats were recorded in real time (sample rate: 384 kHz, 16 bit) using a bat detector (D1000X; Pettersson Elektronik AB, Uppsala, Sweden). Recordings were triggered manually while listening through headphones to the output of the heterodyne system of the detector and continuously scanning the frequency range from 20 to 80 kHz. As the heterodyne mode of the Pettersson system covers a 10 kHz frequency band around the respective scanning frequencies, we were able to detect all locally occurring bat species and trigger a sound recording. The pretrigger time was set at 10 sec and the post‐trigger time at 50 sec which resulted in a standardized file length of one‐minute recordings.

### Analysis of acoustic data

We used Avisoft‐SASLab Pro, version 4.53 (Avisoft Bioacoustics, Berlin, Germany) to analyze the acoustic recordings. Spectrograms were created using a Hamming window (512 FFT, 93.75% overlap). Activity of bats was determined by counting the number of passes per minute and plot. Hereby, a pass was defined as a sequence of at least two consecutive echolocation pulses of one individual bat (Fenton 2004). Successive passes within one‐minute files were considered as separate passes if the time interval exceeded three times the regular pulse interval of the respective species (Estrada‐Villegas et al. 2010). We also assessed feeding attempts by counting the number of terminal phases. Terminal phases are characterized by a characteristic increase in echolocation call emission rate prior to prey capture attempts (Kalko and Schnitzler 1998). We considered the number of feeding attempts as a direct measure of a bat–insect interaction.

Echolocation sequences were identified to species or sonotype by analyzing call structure, start, peak, and end frequency, following an own custom‐made identification key and data from previous publications (e.g., Russo and Jones 2002; Skiba 2003; Obrist et al. 2004). We unambiguously identified echolocation sequences of *Pipistrellus nathusii, Pipistrellus pipistrellus*,* Pipistrellus pygmaeus*,* Nyctalus noctula*,* Nyctalus leisleri*,* Eptesicus nilssonii,* and *Myotis myotis*. We are aware that *Eptesicus nilssonii* is considered rare in Brandenburg; however, we recorded echolocation calls with end frequencies from 27 to 31 kHz typical for this species (Rydell 1993). All other identified species are known to frequently occur in the region of Schorfheide‐Chorin (Teubner et al. 2008). Several species which are difficult to discriminate acoustically were grouped into sonotypes. We did not discriminate between *Plecotus auritus* and *P. austriacus* and grouped them to *Plecotus* spec. (0.3% of our data). Finally, we assigned echolocation sequences with very similar echolocation call structure and frequency (11.7% of our data) to the sonotypes *Nyctaloid* low, *Nyctaloid* high, and *Myotis spec*. (for details, please refer to the Supporting Information, and Heim et al. 2015). We then calculated the effective number of species, e^H^ (Jost 2006), to estimate bat species diversity on grassland sites.

### Insect sampling

Parallel to the 24‐min acoustic monitoring of bats per grassland site, we collected nocturnal insects with flight interception light traps (super actinic 12 V, 8 W; wavelength: 300–460 nm). Except for the order Lepidoptera, which were frozen, the captured insects were preserved in 70% nondenaturized alcohol. We assessed the number of individuals per sample, measured body size, and identified them to order (following Chinery 2002). To estimate differences in insect diversity between grassland sites, we calculated the effective number of insect orders (Jost 2006). In addition, we computed the proportional size and the proportional diversity of insects per site by dividing both measures through the number of captured individuals. We did this, as we assumed that the proportional size and diversity of prey items should be most important for bats (Safi and Siemers 2010).

### Local and landscape variables

Local land‐use intensity of grassland sites was quantified using a standardized land‐use intensity index (LUI). This index is based on an annual questionnaire to the local land user and it is measured by the sum of the regionally standardized intensity of the four main management components, frequency of mowing, livestock unit, grazing days per hectare, and fertilization in kg nitrogen per hectare (Blüthgen et al. 2012).

The direct surrounding landscape of each recording site was assessed based on a digital landscape model (Version 2009, resolution: 1:10,000, Landesvermessungsamt Brandenburg) and aerial photographs of the region (taken in 2009) using ArcGIS 9.31 (ESRI, Redlands, CA). Hereby, we used the distance to the nearest forest patch as a proxy for site accessibility. This measure has shown to be highly important for bat activity and species richness in a previous study within the same region (Heim et al. 2015).

### Data analysis

We assessed the effect of land‐use intensity and site accessibility on insects and bats using generalized linear mixed effect models (glmer, package lme4). For insects, we calculated three models with abundance, proportional size, and proportional diversity as the response variables. All models included land‐use intensity quantified through the LUI index and distance to the nearest forest patch of each grassland site as fixed factors. Land‐use intensity was nested within soil types, as the effect of land‐use intensity might differ due to soil conditions (Stohlgren et al. 1999).

For bats, we calculated separate models for activity, feeding attempts, and species diversity as response variables. For all models, we used Poisson distribution as data originated from counts. Noninteger numbers were rounded in its first argument. In all models for bats, we included number of captured insects, proportional size, and proportional diversity of insects as additional explanatory variables. Grassland sites were included as random factors to account for repeated sampling during the season.

In addition, we examined whether the response of feeding attempts on sites with different land‐use intensity varied with the distance to forested areas. For this, we divided our sample into categories of equal N halves (50%) of higher (range 1.45–3.09) and lower (range 0.75–1.44) land‐use intensity (following Manning et al. 2015) and, respectively, in closer (range: 40–160 m) and further (range 160–500 m) distance to the nearest forest patch. Both categories were then included as interacting factors into the model concerning bat feeding attempts, and sampling sites were kept as a random factor. Additionally, an analysis of deviance (type II Wald chi‐square tests) was used as a post hoc test.

Nonmetric multidimensional scaling (NMDS package: vegan R, Oksanen et al. 2008) was used to investigate differences in species composition of bats between grassland plots. We further conducted a permutated multivariate analysis of variance (Adonis, package: vegan) to evaluate the effects of land‐use intensity and distance to forest on bat species and insect order composition. Finally, we performed a mantel test (package: vegan R) on distance matrices of bats and insects between grassland plots (Legendre & Legendre 1998) to test whether differences in species composition of bats correlate with differences in the composition of insect orders.

All statistical tests were conducted using R statistical package version 2.13.1 (R Development Core Team 2011).

## Results

As expected, insects responded predominantly to local land‐use intensity and soil conditions. Insect size significantly decreased with higher land‐use intensity. On fen soils, the number of insects significantly increased with increasing land‐use intensity (Table 1).

**Table 1:** Effect of local land‐use intensity and distance to forested areas on insect abundance, size, and diversity of insect orders on grassland sites of the Schorfheide‐Chorin. Intercepts are presented in italic font; n.s. refers to non‐significant results

Insects	Parameters	Estimate	SE	*Z*	*P* < *z*
Abundance deviance: 4145.7	*Intercept*	*−0.06*	*0.69*	*−0.08*	*<0.001*
	LUI	*−*0.27	0.46	*−0.59*	*n.s*.
	LUI on fen soil	0.84	0.31	2.75	<0.01
	Distance to forest	<0.01	<0.01	*0.45*	*n.s*.
Proportional size deviance: 599.0	*Intercept*	*0.35*	*<0.01*	*172.62*	*<0.001*
	LUI	*−*0.17	0.01	*−*85.60	*<0.001*
	LUI on fen soil	*−*0.11	0.01	*−*52.90	*<0.001*
	Distance to forest	*−*0.00	0.00	*−0.82*	*n.s*.
Proportional order diversity deviance: 231.6	*Intercept*	*−1.15*	*0.53*	*−2.17*	*0.05*
	LUI	*−*0.15	0.34	*−0.44*	*n.s*.
	LUI on fen soil	<*−*0.01	0.22	*−0.01*	*n.s*.
	Distance to forest	<*−*0.01	<0.01	*−0.09*	*n.s*.

Bats, in contrast, were predominantly influenced by the proximity to forest patches, rather than the local land‐use intensity. Bat activity and diversity increased significantly in proximity to forested areas. Activity of bats also increased with higher insect abundance and proportional diversity of insect orders, but decreased with proportional insect size. In addition, bat diversity responded to grassland management and decreased with increasing land‐use intensity (Table 2).

**Table 2:** Response of bat activity, diversity, and feeding attempts to local land‐use intensity, distance to forested areas and resource availability (measured in abundance, size, and diversity). Intercepts are presented in italic font; n.s. refers to non‐significant results

Bats	Parameters	Estimate	SE	*Z*	*P* < *z*
Activity deviance: 7679.2	*Intercept*	*4.33*	*0.39*	*11.11*	*<0.001*
	Insect abundance	*<0.01*	*<0.01*	*8.33*	*<0.001*
	Proportional size of insects	*−0.79*	*0.02*	*−35.14*	*<0.001*
	Proportional insect diversity	*0.31*	*0.05*	*6.37*	*<0.001*
	LUI	*−0.14*	*0.21*	*−0.67*	*n.s*.
	Distance to forest	*<−0.01*	*<−0.01*	*−3.02*	*<0.01*
Diversity deviance: 725.5	*Intercept*	*1.50*	*0.15*	*10.20*	*<0.001*
	Insect abundance	<0.01	<0.01	0.17	n.s.
	Proportional size of insects	*−*0.15	0.05	*−*3.3	<0.01
	Proportional insect diversity	*−*0.03	0.13	*−*0.19	n.s.
	LUI	*−0.17*	*0.07*	*−2.10*	*<0.05*
	Distance to forest	*<−0.01*	*<0.01*	*−3.3*	<0.001
Feeding attempts deviance: 2329.0	*Intercept*	*2.35*	*0.57*	*4.09*	*<0.001*
	Insect abundance	0.01	<0.01	2.63	<0.01
	Proportional size of insects	*−*1.50	0.06	*−*22.16	<0.001
	Proportional insect diversity	0.55	0.14	4.01	<0.001
	LUI	*−*0.07	0.34	0.21	n.s.
	Distance to forest	<*−*0.01	<0.01	*−*2.43	<0.01

Feeding attempts of bats increased significantly with higher numbers of insects and diversity of insect orders but decreased with proportional insect size. Furthermore, feeding attempts significantly decreased further away from forested areas (Table 2).

**Figure 1:**
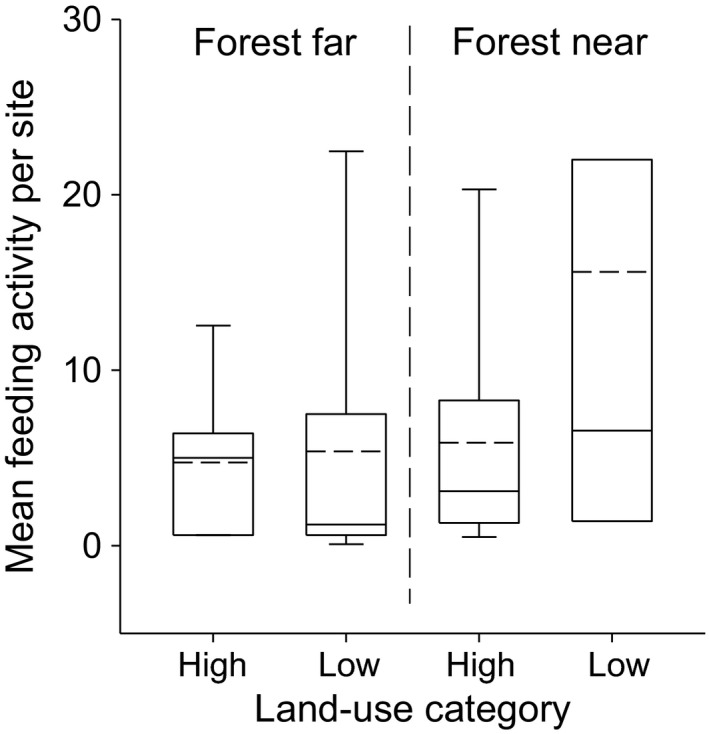
Mean feeding attempts of bats on grassland site categories differing in land‐use intensity and distance to forested areas. Median is depicted as continuous line and the mean as dashed line.

Contrasting land‐use intensity and forest distance classes underlined that an interaction of local land‐use intensity and distance to forest affected the predator–prey relationship between bats and insects (Table 3). Near forested areas, the number of feeding attempts was much lower on grassland sites with high, compared to low land‐use intensity (Fig. 1). In contrast, far from forests, feeding activity did not differ between intensively and extensively managed grassland sites.

**Table 3:** Interacting effect of land‐use intensity and forest distance categories on the predator–prey interaction of bats and insects measured in feeding attempts

Feeding attempts	Parameters	Chi‐square	*P*
	LUI category	0.3527	n.s.
	Forest category	7.3946	<0.01
	LUI category × forest categories	3.8939	<0.05

**Figure 2:**
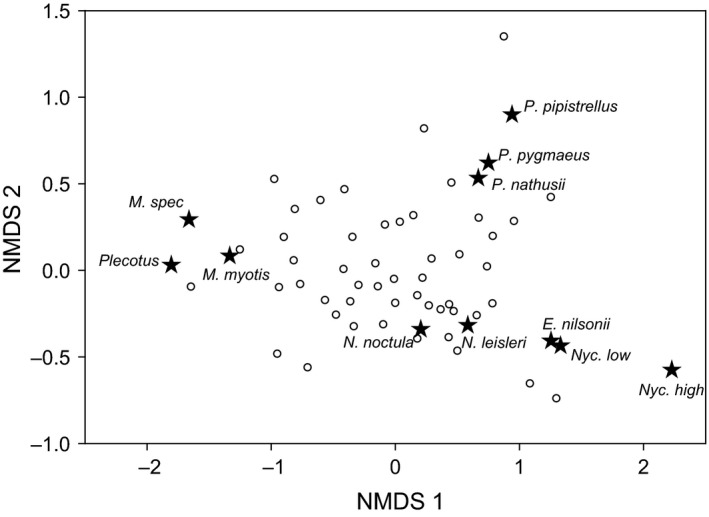
Nonmetric multidimensional scaling of grassland plots based on Bray–Curtis dissimilarity of bat activity. Grassland plots are displayed as circles and bat species are depicted as asterisks.

Nonmetric dimensional scaling significantly separated grassland sites based on differences in species composition of bats (NMDS, final stress = 0.096, linear fit *r* ^2^ = 0.962, Fig. 2). Hereby, grassland sites differed most evidently in the relative activity of species that can be classified into different foraging guilds. NMDS axis 1 clearly separated sites with a higher activity of narrow‐space foragers such as the gleaning genera *Myotis* and *Plecotus* from sites with higher activity of edge foragers (*Pipistrellus)* and open‐space foragers (*Nyctalus/Eptesicus*). NMDS axis 2 mostly separated grassland sites based on differences in activity between edge‐ and open‐space foragers (Fig. 2).

Differences in the composition of bat species (*F*_(1,49)_ = 3.5, *P* < 0.01) and insect orders (*F*_(1,49)_ = 2.5, *P* < 0.05) were significantly explained by distances to forest patches, while local land‐use intensity revealed no significant influence neither on bats (*F*_(1,49)_ = 0.8, *P* > 0.05) nor on insects (*F*_(1,49)_ = 1.3, *P* > 0.05). However, differences in bat species composition between grassland sites did not correlate with differences in the composition of insect orders (Mantel statistic, *r* = 0.01, *P* > 0.05), suggesting distinct driving factors determining species composition of both taxa.

## Discussion

Our results confirmed that bats and nocturnal insects respond on different scales to changes in their environment. While insects were influenced by local land‐use and soil conditions, bat activity and diversity predominantly responded to differences in site accessibility of the surrounding landscape matrix, measured in distance to forested areas.

Both local land‐use and soil conditions in combination are causing differences in local plant diversity and vegetation complexity (Stohlgren et al. 1999), which in turn are important driving factors for insect diversity and abundance (Haddad et al. 2000; Reid and Hochuli 2007; Socher et al. 2012). Our results show that increased land‐use intensity leads to a higher abundance and a higher proportion of smaller insects. This is in agreement with Simons et al. (2015), who showed that increasing land‐use intensity causes an increased abundance of smaller sized day‐active insects besides affecting species composition of insect assemblages.

Bat activity, diversity, and species composition were predominantly determined by the distance to the nearest forest area and thus by accessibility of grassland sites. This is in agreement with several previous publications documenting the high importance of forested areas on bat activity and species composition (Frey‐Ehrenbold et al. 2013; Kalda et al. 2014; Heim et al. 2015).

Thus, our results clearly show, in agreement with previous publications (Söderström et al. 2001; Pocock and Jennings 2008), that invertebrates are more sensitive to agricultural intensification compared to insectivorous mammals, which are rather sensitive to losses in landscape heterogeneity including reduced forest cover and hedges or tree lines, which could serve as stepping stones and thus assure site accessibility within agricultural landscapes (Fuentes‐Montemayor et al. 2011).

Insect abundance increased with higher land‐use intensity on fen soils, and feeding attempts of bats increased with higher insect abundance. However, feeding attempts and bat diversity decreased further away from forested areas. This indicates that the control of insect populations by bats decreases on grassland sites far away from forested areas and, in addition, is carried out by only a few bat species (e.g., *N. noctula*). In contrast, grassland sites at close proximity to forests profited from an increase in feeding attempts of many different bat species.

Our results also showed that near forested areas, feeding attempts decreased significantly on grassland site with high, compared to those with low land‐use intensity. We argue that this is caused by an indirect effect of land‐use intensity, via reduced availability of insects for bats. Although the number of insects increased with land‐use intensity, insect size and diversity decreased. Bats generally can, even within a single night, explore a large area for suitable foraging sites. This flexibility allows them to quickly respond to changes in resource density (McCracken et al. 2012), which might vary due to local land use. Thus, our results suggest that near forests, bats escaped unfavorable conditions and rather foraged at extensively managed grassland sites harboring higher diversity of generally larger insects. This agrees with the hypothesis that higher mobility and larger activity ranges enhance the possibility of organisms to cope with local disturbances (e.g., Leibold et al. 2004; Öckinger et al. 2010; Concepción et al. 2015).

At greater distance to forests, feeding attempts of bats decreased and intensively managed grasslands did not differ from extensively managed grasslands. This goes in concert with a reduction in general activity of bats and underlines the importance of site accessibility for bats. Far away from forest, observed feeding attempts mainly originated from fast‐flying open‐space foragers such as *N. noctula*. This species uses echolocation calls at rather low frequencies (19/21 kHz) which are well suited for long‐range detection of larger prey items (Safi and Siemers 2010; Jakobsen et al. 2013; Jung et al. 2014), but are not optimal for the detection and successful capture of smaller prey during fast flight. Meanwhile, narrow‐ and edge‐space foragers, calling at higher frequencies adapted to detect smaller insects, are unable to access such potential food sources due to an insufficiently connected landscape (Fuentes‐Montemayor et al. 2011). This suggests an ecological mismatch of available insect prey and the presence of predator species with increasing land‐use intensity and loss of forested areas.

We conclude that forested areas are an important prerequisite for higher bat species diversity and feeding activity within agricultural landscapes. However, forest proximity of grassland sites alone is not sufficient to compensate for local high land‐use intensity. We thus argue that land‐use intensification may affect individual taxa at very different scales; however, a combination of local‐ and landscape‐scale effects can affect cross‐taxa interactions and thus cause a loss in local ecosystem function.

## Conflict of Interest

None declared.

## Supporting information


**Table S1.** Echolocation call characteristics of bat species and sonotypes, used for the identification of recorded sound sequences in the Schorfheide‐Chorin.
