# Supplementary material for: The effect of local land use and loss of forests on bats and nocturnal insects
Source: Ecol Evol. 2016 May 27;6(13):4289–97. doi: 10.1002/ece3.2160 (PMC4930980; doi:10.1002/ece3.2160)
Supplement: Supplementary file 1 — Table S1. Echolocation call characteristics of bat species and sonotypes, used for the identification of recorded sound sequences in the Schorfheide‐Chorin. [file ECE3-6-4289-s001.doc]

S-Table 1: Echolocation call characteristics of bat species and sonotypes, used for the identification of recorded sound sequences in the Schorfheide-Chorin. Peak frequency is given in kHz and additional characteristics used for species identification are listed under call characteristics.

| Species | Foraging space | kHz | Call characteristics |
| --- | --- | --- | --- |
| *Nyctalus noctula* | open | 18/21 | Quasi-constant frequency call, regular call frequency alternation |
| *Nyctalus leisleri* | open | 22/24 | Quasi-constant frequency call, call alternation |
| *Eptesicus nilssonii* | open | > 27 | Quasi-constant frequency call, conspicuous second harmonic |
| Sonotype: *Nyctaloid low* | open | 22-25 | Quasi-constant frequency call, potentially including: *N. leisleri, Vespertilio murinus* and *Eptesicus serotinus* |
| Sonotype: *Nyctaloid high* | open | 25-27 | Quasi-constant frequency call, potentially including: *N. leisleri, E. serotinus* and *E. nilssonii* |
| *Pipistrellus nathusii* | edge | 39-40 | Frequency modulated call with quasi constant frequency component |
| *Pipistrellus pipistrellus* | edge | 45 | Frequency modulated call with quasi constant frequency component |
| *Pipistrellus pygmaeus* | edge | 53 | Frequency modulated call with quasi constant frequency component |
| *Myotis myotis* | narrow | 25-35 | Frequency modulated call, call duration > 5ms, and pulse interval > 100ms |
| *Myotis* spec. | narrow | 35 | Frequency modulated call, potentially including *M. nattererii,* *M. bechsteinii, M. mystacinus, M. brandtii, M. dascyneme, M. daubentonii* |
| *Plecotus* spec. | narrow | 22 | Frequency modulated, multi-harmonic call, potentially including: *P. auritus* and *P. austriacus*, terminal frequency very low < 15 kHz |
